# Red urine in a 68‐year‐old man with amyotrophic lateral sclerosis

**DOI:** 10.1002/jgf2.70066

**Published:** 2025-09-18

**Authors:** Kento Furuya, Naoya Itoh

**Affiliations:** ^1^ Department of Internal Medicine Izu Redcross Hospital Shizuoka Japan; ^2^ Department of Infectious Diseases, Graduate School of Medical Sciences Nagoya City University Aichi Japan; ^3^ Department of Infectious Diseases Nagoya City University East Medical Center Aichi Japan; ^4^ Department of Clinical Infectious Diseases, Graduate School of Medical Sciences Nagoya City University Aichi Japan

**Keywords:** amyotrophic lateral sclesosis, methylcobalamin, red uirne

A 68‐year‐old man was diagnosed with amyotrophic lateral sclerosis (ALS) 2 years ago. He had been previously treated with edaravone and riluzole. We initiated high‐dose methylcobalamin (50 mg, twice weekly by intramuscular injection) as part of home visit medical care. The day after starting methylcobalamin, his urine turned red (Figure 1A). At that time, he had no symptoms of abdominal or urinary pain. A dipstick test was negative for blood, urobilinogen, and bilirubin, and urine sedimentation tests showed no erythrocytes. Urine myoglobin was also negative. Three days after the methylcobalamin treatment, his urine color returned to yellow (Figure 1B). Following each subsequent dose of methylcobalamin, his urine again turned red. Based on these findings, we concluded that the change in urine color was caused by methylcobalamin. Methylcobalamin has long been used to treat peripheral neuropathy.^1^ High doses of methylcobalamin are effective in slowing disease progression in patients with early ALS.^2^ The occurrence of red urine during high‐dose vitamin B12 therapy has been documented in two published clinical trials.^2^, ^3^ The occurrence of reddish urine has been noted in the package insert of the vitamin B12 formulation indicated for ALS.^4^ However, patients may become alarmed upon noticing reddish urine. Therefore, it is important to inform them of this possibility in advance. Additionally, red urine can result from various medications (e.g., rifampin, chloroquine), intravascular hemolysis (e.g., hemolytic anemia, G6PD deficiency), other medical conditions (e.g., nephrolithiasis, nutcracker syndrome), or dietary factors (e.g., beets, blackberries).^5^ Thus, if red urine occurs during methylcobalamin administration, other potential causes should be carefully ruled out.

**FIGURE 1:**
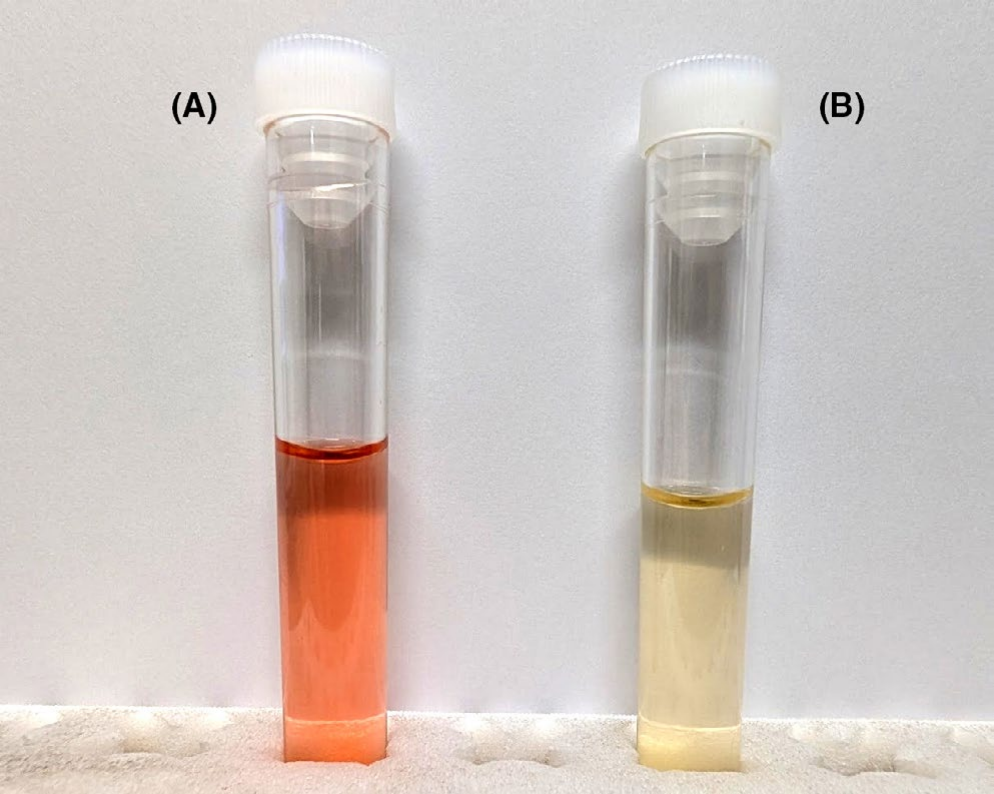
(A) Urine on the day after high‐dose methylcobalamin administration. (B) Urine 3 days after high‐dose methylcobalamin administration.

## AUTHOR CONTRIBUTIONS

**Kento Furuya:** conceptualization; writing—original draft preparation; writing—review and editing (lead). **Naoya Itoh:** Funding acquisition; supervision; writing—review and editing (supporting).

## FUNDING INFORMATION

None.

## CONFLICT OF INTEREST STATEMENT

None declared.

## ETHICS STATEMENT

None.

## PATIENT CONSENT

The patient has provided written consent for the publication of the images and the accompanying text.

## Data Availability

The data that support the findings of this study are available from the corresponding author upon reasonable request.
